# Mechanochemical Strategies for the Preparation of SiO_2_-Supported AgAu Nanoalloy Catalysts

**DOI:** 10.3389/fchem.2022.836597

**Published:** 2022-02-02

**Authors:** Rafael T. P. da Silva, Susana I. Córdoba De Torresi, Paulo F. M. de Oliveira

**Affiliations:** Institute of Chemistry, University of São Paulo - USP, São Paulo, Brazil

**Keywords:** mechanochemical synthesis, bottom-up approach, ball-milling, bimetallic nanoparticle, nanoalloys, nanocatalysis, galvanic replacement reaction, nitrobenzene reduction

## Abstract

Silver-gold nanoalloys were prepared from their metal salts precursors through bottom-up mechanochemical synthesis, using one-pot or galvanic replacement reaction strategies. The nanostructures were prepared over amorphous SiO_2_ as an inert supporting material, facilitating their stabilization without the use of any stabilizing agent. The nanomaterials were extensively characterized, confirming the formation of the bimetallic nanostructures. The nanoalloys were tested as catalysts in the hydrogenation of 2-nitroaniline and exhibited up to 4-fold the rate constant and up to 37% increased conversion compared to the respective single metal nanoparticles. Our approach is advantageous to produce nanoparticles with clean surfaces with available catalytic sites, directly in the solid-state and in an environmentally friendly manner.

## 1 Introduction

The interest in designing metal nanoparticles (NPs) is in constant increase, resulting from the exceptional properties of the nanostructures, which are relevant in catalysis (Araujo et al., 2019), energy (Califano et al., 2021), biomedicine (Li et al., 2017), and sensing (Jeon et al., 2016; Ma et al., 2020). Many advances have been made in tuning the properties of single metal NPs by controlling their size and shape. Nonetheless, a material containing a single element hardly satisfies all the requirements for a certain application. In catalysis field, for example, an ideal material should be highly active, selective, chemically, and structurally stable. Additionally, for industrial purposes the material should also be available at scale. These technological demands can be accomplished by the association of two or more elements into the final nanostructure. The combination of different metals in a single nanostructure is an opportunity to integrate the properties emerging from all individual contributors in a synergistic fashion (Gilroy et al., 2016).

Solution-based and thermal-annealing protocols have succeeded in the preparation of multimetallic structures containing two, eventually three elements (da Silva et al., 2016; Bhunia et al., 2018; Quiroz et al., 2018; Rodrigues et al., 2021). The solution-based techniques are, however, considerably sensitive to the reaction conditions, and are solvent- and/or energy-intensive. In addition, classical routes still face challenges for the preparation of nanostructures at larger scales (Paliwal et al., 2014). In the past decades, environmentally friendly syntheses have been developed in order to overcome these issues, including the use of bio-based reducing agents and alternative energy inputs (Iravani, 2011; Baláž et al., 2017; Ahmad et al., 2019; Jorge de Souza et al., 2019). In such contexts, mechanochemistry and mechanically-induced transformations in the solid-state (Boldyrev, 2006; Boldyreva, 2013) have gained prominence as an alternative to solvent-based protocols in the preparation of a diversity of chemicals (James et al., 2012) and materials (Xu et al., 2015; Muñoz-Batista et al., 2018). In fact, the use of mechanical energy to induce transformations in multimetallic systems through milling, the so-called mechanical alloying (Suryanarayana, 2001), is one of the its most explored applications. The early works were mostly dedicated to investigate the alloying of non-noble transition metals via top-down approaches, i.e., by milling the elemental powders (Suryanarayana, 2001).

More recently, noble metal nanoparticles have been more investigated under mechanochemical conditions, however, similarly to previous works, the studies have used the top-down methodology with elements such as Au, Ag and Pd to produce the final multicomponent nanomaterials (Delogu, 2008; Danielis et al., 2018; Schreyer et al., 2019; De Bellis et al., 2021). The greatest advantage offered by the technique is related to the simplicity of the treatment, dismissing any post-synthetic workup. On the other hand, top-down approaches are limited in terms of size and shape control. The alternative is to start from the metal salt precursors and construct the nanostructure by the chemical reduction or decomposition, known as bottom-up approach (Moores, 2018; de Oliveira et al., 2020b). The reduction or decomposition processes enable the control of reaction rate, nucleation and generation of growing species, leading to nanoparticles with tunable sizes and shape. Previous works have demonstrated that mechanochemical synthesis in ball milling devices is a promising strategy for the preparation of catalytically relevant noble single metal nanoparticles from their metal precursors (Debnath et al., 2009; Rak et al., 2014; De Oliveira et al., 2019; de Oliveira et al., 2020a; Baláž et al., 2021; Jin et al., 2021; Quiroz et al., 2021). On the other hand, few works associate more than one metal in the final structure totally through bottom-up mechanochemical synthesis. The examples include Pd-Ag nanoalloys (Ruiz-Ruiz et al., 2018, 2022), and Ag-Au nanoclusters (Murugadoss et al., 2012).

In the present report, we describe different approaches for the bottom-up mechanochemical synthesis of noble metal AgAu nanoalloys. Ag and Au were chosen due to their high miscibility and the possibility of a galvanic replacement according to the standard redox potentials of Ag and Au species. Additionally, Ag and Au NPs displays their localized surface plasmon resonance (LSPR) in the visible range, making the alloyed NPs suitable candidates for further studies as plasmonic materials (Liu et al., 2018). For the preparation of the nanoalloys, we adopted two strategies; 1) one-pot/co-reduction of Ag and Au metallic precursors and, 2) two-step galvanic replacement reaction (GRR), using Ag NPs as templates. The use of amorphous SiO_2_ as solid support enabled the stabilization of the nanoalloys in sizes smaller than 10 nm without using any surfactant or capping agent. The clean surfaces are an advantage for catalytic applications. Thus, the final nanoalloys were tested in the catalytic reduction of 2-nitroaniline (2-NA) to *o*-phenylenediamine (*o*-PDA), and exhibited superior activities compared to their monometallic Ag and Au NPs counterparts.

## 2 Materials and Methods

### 2.1 Chemicals

Flash silica (high-purity grade, Sigma-Aldrich), HAuCl_4_.3H_2_O (hydrogen tetrachloroaurate trihydrate, 48% in gold, Sigma-Aldrich), AgNO_3_ (silver nitrate, ≥99%, Sigma-Aldrich), C_6_H_6_N_2_O_2_ (2-nitroaniline, 98%, Sigma-Aldrich), C_6_H_8_N_2_ (o-phenylenediamine, ≥99%, Sigma-Aldrich), NaBH_4_ (sodium borohydride, powder, ≥98%, Sigma-Aldrich). All chemicals were analytical grade reagents. Deionized water (18.2 MΩ) was used throughout the experiments.

### 2.2 Instrumentation

Transmission electron microscopy (TEM) and high-resolution TEM (HRTEM) images were obtained using a JEOL JEM 2100 operated at 200 kV. A Talos F200S microscope (Thermo Scientific) operating at 200 kV was used for high-angle annular dark-field imaging scanning transmission electron microscopy (HAADF-STEM) and STEM-energy dispersive x-ray analysis (STEM-EDX). Samples were prepared by drop casting 5 μL from a well dispersed isopropanol suspension of each sample on a Formvar/Carbon Film coated, 200 Mesh, copper grid. UV-VIS and diffusive reflectance spectra (DRS) of the solid samples were obtained using a Shimadzu UV-2600 UV-VIS spectrophotometer. For catalytic experiments in water a quartz cuvette with an optical path of 1 cm was used. Powder X-ray diffraction (XRD) patterns were recorded in Shimadzu 7,000 Maxima diffractometer with Cu Kα radiation (λ_CuKα_ = 1.5418 Å for combined Kα_1_ and Kα_2_) in a Bragg-Brentano geometry. XPS analyses were performed with a UNI-SPECS UHV at pressure below 5·10^–7^ Pa (Mg_Kα_ hν = 1,253.6 eV). A carbon tape was used as substrate to fix the samples. To determine the metal composition in the samples, inductively coupled plasma–optical emission spectrometry (ICP-OES) Spectro Arcos equipment was used. Aqua regia or HNO_3_ was used for the digestion of the samples. Then, a 10-fold dilution with distilled water was performed before each measurement. Further details can be found in the Supplementary Material.

### 2.3 Bottom-Up Mechanochemical Synthesis of Supported Metal Nanoparticles

#### 2.3.1 Silver Nanoparticles–Ag/SiO_2_

Typically, Ag NPs/SiO_2_ were synthesized using AgNO_3_ (19.6 mg) as precursor and NaBH_4_ (19.6 mg) as reducing agent in presence on SiO_2_ (250 mg). The reactants and the support were placed in a PMMA jar containing one single ZrO_2_ milling ball (ø = 10 mm, m = 3.14 g) and milled at 50 Hz for 60 min in a vibratory ball-mill Pulversitte 23 Fritsch®. After the milling period the powder underwent several cycles of redispersion in water and centrifugation to eliminate the byproducts formed.

#### 2.3.2 Gold Nanoparticles–Au/SiO_2_

Au/SiO_2_ nanoparticles were prepared similarly to Ag/SiO_2_. HAuCl_4_.3H_2_O (25 mg) was used as gold precursor and NaBH_4_ (25 mg) as reducing agent. SiO_2_ (250 mg) was utilized as solid support.

#### 2.3.3 AgAu Nanoalloy - Galvanic Replacement Reaction

HAuCl_4_.3H_2_O (7.5 mg) and cleaned and dried AgNPs/SiO_2_ (250 mg) were added to the milling jar and milled for 60 min at 50 Hz. Note that no reducing agent was used and the Au^3+^ reduction to metallic gold was induced only by the galvanic replacement of Ag^0^ according to Eq. 1. To eliminate the byproduct, i.e., AgCl, the powder was dispersed in a supersaturated NaCl solution, to generate (AgCl_x_)^−(x−1)^ (x > 1) soluble species, and centrifuged. A final washing cycle was performed with water.

$$AuCl_{4}^{-}+Ag^{0}\to 3Ag^{+}+\;Au^{0}+4Cl^{-}\;\;\;\;\varepsilon^{0}=0.2\;V$$ (1)

#### 2.3.4 AgAu Nanoalloy - One-Pot Synthesis

The one-pot synthesis of AgAu NAs was performed by the simultaneously addition of AgNO_3_ (9.8 mg). HAuCl_4_.3H_2_O (12.5 mg) (Ag: Au ratio 1:1 m/m) and NaBH_4_ (22.3 mg) into the milling jar containing SiO_2_ (250 mg).

## 2.4 Catalytic Reduction of 2-Nitroaniline

Hydrogenation of 2-nitroaniline (0.75 μmol) were performed with NaBH_4_ (0.26 μmol) in triplicates in a quartz cuvette and monitored using a UV-Vis spectrophotometer ranging from 500 to 250 nm. The total load of metal used was 1.6 *wt*%, considering the catalyst/substrate ratio. (Details in the Supplementary Material).

## 3 Results and Discussion

### 3.1 Materials Synthesis and Characterization

The specific combination of Ag and Au as noble metals in bimetallic systems offers the opportunity to minimize the cost of Au catalysts and, at the same time, enhance the material catalytic performance through use synergistic effects (Slater et al., 2014). Typically, Ag and Au are combined either in core-shell or in alloys to applications in biosensors (Jia et al., 2014), SERS (Chen et al., 2013; Girão et al., 2017), catalysis (Wang et al., 2014; Kisukuri et al., 2016; Rodrigues et al., 2016) or electrocatalysis (Nazir et al., 2017; Balasubramanian et al., 2020; da Silva et al., 2021). They may be either blended in nanocrystals comprised of the individual elements or in an alloyed or intermetallic atomic ordering (Gilroy et al., 2016). Most of the experimental protocols requires capping agents to keep the colloidal nanoparticle dispersion stable. Capping agents, however, usually act as a physical barrier hindering the catalytic surface of nanoparticles from the access of reactants. In addition, they can act poisoning the catalytic sites (Niu and Li, 2014).

**FIGURE 1:**
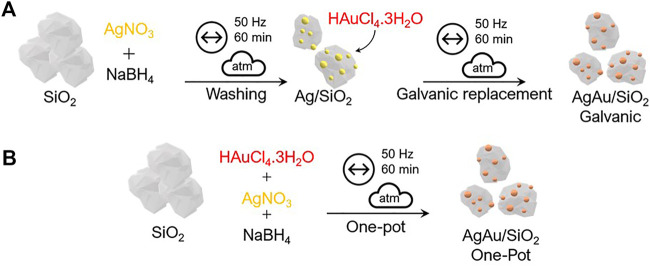
Scheme of the bottom-up mechanochemical synthesis routes for the preparation of AgAu/SiO_2_ galvanic **(A)** and AgAu/SiO_2_ one-pot **(B)** nanoalloys. The symbols over the arrows follow the recommendation of ref. (Michalchuk et al., 2021).

In the present work, two different approaches were used to synthesize AgAu bimetallic alloys supported over amorphous SiO_2_, without any addition of capping agents, as depicted in Figure 1. The first (Figure 1A) is a two-step reaction, where the first step led to the reduction of Ag NPs on amorphous SiO_2_ through the addition of Ag precursor and NaBH_4_ as the reducing agent, leading to Ag/SiO_2_. After washing steps to remove remaining NaBH_4_ and other byproducts, the Ag NPs acted as templates for the mechanochemical GRR. In this second step, due to the difference in the reduction potential of AuCl_4_^−^ and Ag^0^ (Eq. (1)), the Ag atoms quickly oxidize with the addition of Au precursor salt, driving the formation of the nanoalloys, also supported on SiO_2_, named AgAu/SiO_2_ galvanic. The second strategy (Figure 1B) was based in a one-pot co-reduction, where both Ag and Au precursors salts were added simultaneously with NaBH_4_ as a reducing agent with SiO_2_ in a ball mill, leading to AgAu/SiO_2_ one-pot. Besides, monometallic Ag/SiO_2_ and Au/SiO_2_ nanostructures were prepared for the subsequent comparison of the catalytic activity. The metal content of the final materials was determined by ICP-OES (Supplementary Table S1) and the reaction yield for each metal for 1-h milling is given on Supplementary Table S2.

**FIGURE 2:**
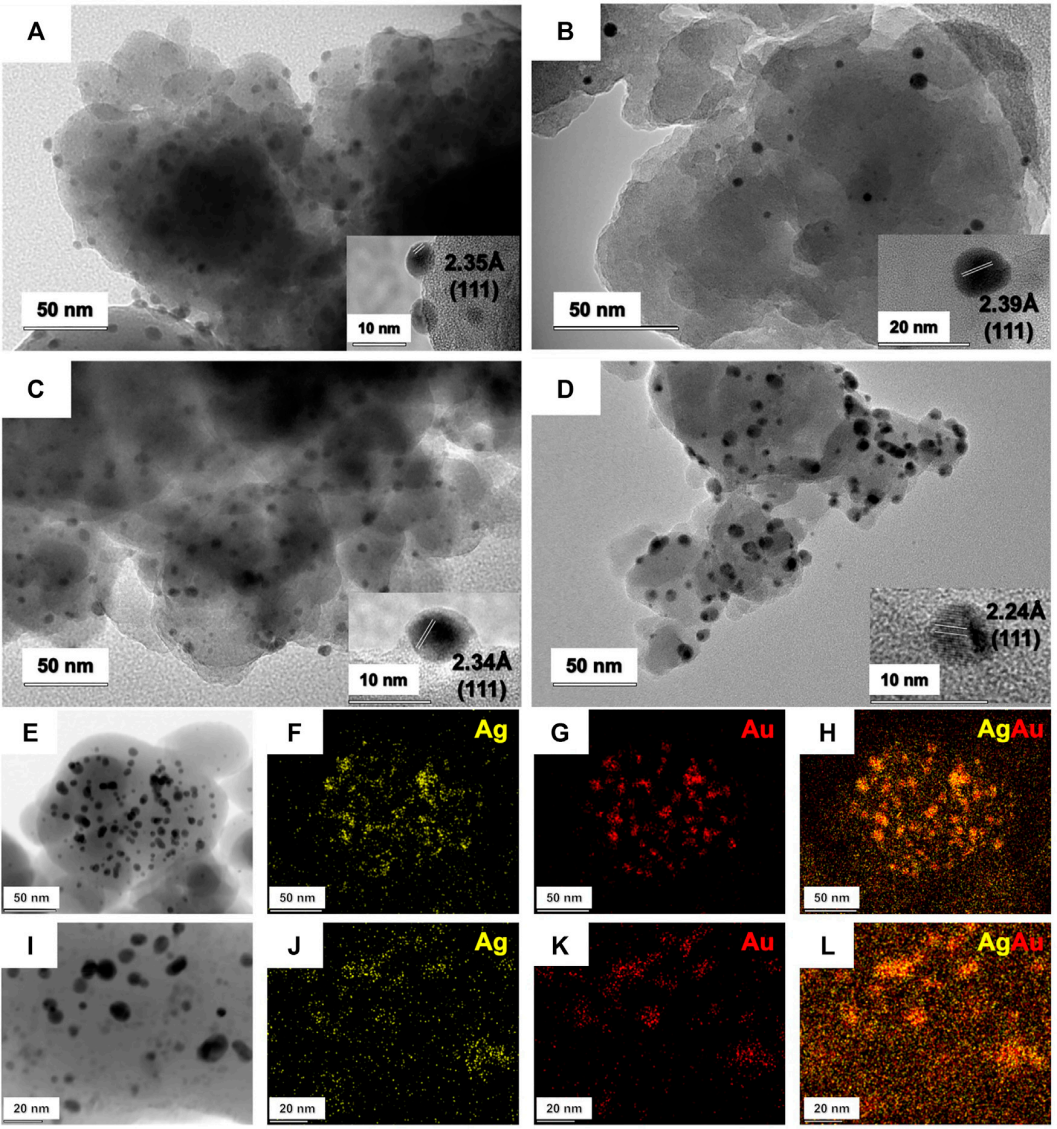
TEM, HRTEM and STEM of the mechanochemically prepared nanoparticles. TEM and HRTEM of AgNP **(A)**, AuNP **(B)**, AgAu/SiO_2_ galvanic **(C)** and AgAu/SiO_2_ one-pot **(D)**. STEM of AgAu/SiO_2_ galvanic **(E–H)** and AgAu/SiO_2_ one-pot **(I–L)**.

TEM micrographs of the synthesized samples are depicted in Figures 2A–D. TEM images show an even distribution of rounded nanoparticles throughout the supporting material. Particle-size distribution for each sample was determined through averaging the diameter of at least 150 nanoparticles, measured one-by-one (Supplementary Figure S1). Single metal Ag/SiO_2_ (Figure 2A) and Au/SiO_2_ (Figure 2B) present relatively polydisperse particle sizes of 5.9 ± 1.7 nm and 6.4 ± 3.3 nm, respectively. On the other hand, the generated bimetallic nanoalloys displayed a relatively narrow size distribution of 6.1 ± 1.7 nm for AgAu/SiO_2_ galvanic (Figure 2C) and 4.0 ± 1.0 nm for AgAu/SiO_2_ one-pot samples (Figure 2B). When comparing the size of the Ag NPs templates for GRR (5.9 nm) and the final bimettalic nanoalloys (6.1 nm), we see no significant difference, which suggests a constant rate of Ag^0^ oxidation and Au^0^ deposition. Interestingly, the one-pot route generated smaller nanoparticles (4.0 nm). We believe that this is probably due to the concurrent reactions that could take place: reduction of Ag^+^, reduction of AuCl_4_^−^, GRR of Ag^0^ by Au^3+^ and redeposition/reduction of displaced Ag^+^. Each one of these reactions can occur in different rates but, at the end, there are a number of nucleation events that hampers further particle growth. It was already demonstrated the bottom-up mechanochemical synthesis of NP may not follow the yet known solution chemistry for the construction of similar architectures, making difficult further discussions over the mechanism of formation of the bimetallic systems at this stage (de Oliveira et al., 2020a).

The insets of Figures 2A–D show HRTEM and the lattice spacing of the nanocrystals. All distances found were 2.35 Å (Ag/SiO_2_), 2.39 Å (Au/SiO_2_), 2.34 Å (AgAu/SiO_2_ galvanic) and 2.24 Å (AgAu/SiO_2_ one-pot), which are consistent with the (111) facet of metallic fcc Ag and Au (ICSD 44382 and 44362). STEM of Ag and Au in AgAu/SiO_2_ galvanic and AgAu/SiO_2_ one-pot are displayed in Figures 2E–H and Figures 2I–L, respectively. The images show the colocalization of Ag and Au in both nanostructures. This pattern is plausible due to the high miscibility among the two metals (Guisbiers et al., 2016). Supplementary Figure S2 shows HAADF-STEM of Ag, Au, Si and O for AgAu/SiO_2_ galvanic and AgAu/SiO_2_ one-pot.

**FIGURE 3:**
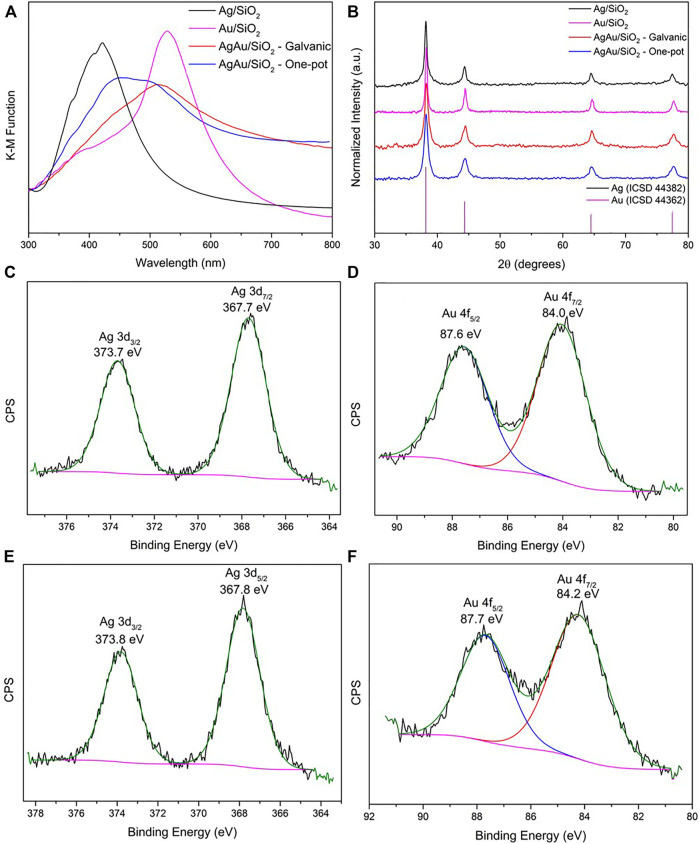
Characterization of the nanoparticles supported on SiO_2_. UV-VIS DRS spectra **(A)**, XRD patterns **(B)** and XPS spectra of AgAu/SiO_2_ galvanic **(C,D)** and AgAu/SiO_2_ one-pot **(E,F)** for the Ag 3d and Au 4f core levels.

UV-VIS diffusive reflectance spectra (DRS) spectra after Kubelka-Munk function of the synthesized materials are presented in Figure 3A. Both monometallic materials present a narrower localized surface plasmon resonance (LSPR) peak in comparison to the bimetallic alloys. LSPR peak referent to Ag/SiO_2_ has its maximum at 422 nm, whereas the maximum of Au/SiO_2_ is at 528 nm, corresponding to the values previously reported for mechanochemical syntheses of these metallic nanoparticles (De Oliveira et al., 2019; Baláž et al., 2021). The bimetallic samples present broader LSPR spectra, composed by overlapped LSPR contributions of shifted Ag and Au NPs and are liable to deconvolution. AgAu/SiO_2_ galvanic LSPR maximum is comprehended at 512 nm and a shoulder peak can be seen at 450 nm. For AgAu/SiO_2_ one-pot, these values are 452 and 501 nm, respectively. This indicates that although there is the formation of alloy through both GRR and one-pot strategies indicated by the redshift in both samples, some remaining monometallic particles could have been formed, as well as regions of higher density of single element within the bimetallic nanostructure. Accordingly, in solution-based synthesis alloyed AgAu nanoparticles present a LSPR broadening and a redshift, varying their maxima starting from the AgNP used as templates to the near infrared (Vongsavat et al., 2011; Gilroy et al., 2016; da Silva et al., 2021).

Regarding the PXRD data presented in Figure 3B, we see that all the samples are crystalline, however displaying a broad diffraction pattern, which indicates the presence of nanoparticles. No significant shifts in the PXRD reflections for the nanoalloys are noteworthy relative to the monometallic Ag/SiO_2_ and Au/SiO_2_ sanples. For example, the reflections relative to (111) crystalline planes represented in 2θ (λ_CuKα_) for single Ag/SiO_2_ and Au/SiO_2_ are 2θ = 38.15° and 38.24°, respectively. Similarly, for bimetallic AgAu/SiO_2_ galvanic and AgAu/SiO_2_ one-pot samples this occurs at 2θ = 38.24° and 38.20°. This is because, both Ag and Au have the same fcc crystal structure and similar lattices constants ( $a_{gold}$ = 4.079 Å; $a_{silver}$ = 4.086 Å) (da Silva et al., 2017). Actually, these parametric similarities facilitate the high miscibility of these metals at nanoscale and promote the formation of uniform AgAu alloys.

To gain further insights in the structure and surface composition of the supported nanoparticles, the samples were also characterized by XPS (Figures 3C–F and Supplementary Figure S3). In the case of single metal Ag/SiO_2_ composite, the XPS spectrum shows two intense photoelectron peaks with binding energies (BE) at the maxima in 368.1 and 374.1 eV, ascribed to Ag 3d_5/2_ and 3d_3/2_ doublet, respectively (Supplementary Figure S3) (Liu et al., 1994). These BE values and the splitting value between them (6.0 eV) are characteristics of metallic Ag, indicating the efficiency of the mechanochemical route in the reduction of the Ag^+^, and that the final material is stable on the support, not undergoing any oxidation when stored in room conditions. Similarly, the XPS spectrum of single metal Au/SiO_2_ demonstrates the formation of metallic Au according to the Au 4f_7/2_ and 4f_5/2_ doublet with BE of 84.1 and 87.6 eV, respectively and a splitting value of 3.5 eV (Supplementary Figure S3) (Mansour, 1994).

Concerning the XPS spectra of the nanoalloys (Figures 3C–F), they demonstrate the metallic character of both metals present in the sample. However, small shifts in the BE can be considered when compared to the respective single metal NPs. In the XPS spectra of AgAu/SiO_2_ galvanic sample (Figure 3C), the peaks related to the Ag 3d_5/2_ and 3d_3/2_ spin orbit coupling doublet, shifts in −0.4 eV while for AgAu/SiO_2_ one-pot sample (Figure 3E) this shift is around −0.3 eV. The shifted BE to lower values is consistent with the formation of a Ag alloyed structure which is, in the present case, with Au (Tyson et al., 1992; Sun et al., 2018). For the BE values of Au 4f_7/2_ and 4f_5/2_ doublet in the samples of alloyed NPs (Figures 3D,F), the shifts compared to Au in the Au/SiO2 sample are meaningless considering the precision of ±0.1 eV in determining the BE.

### 3.2 Catalytic Reduction of 2-Nitroaniline

The successful mechanochemical preparation of bimetallic AgAu nanoalloys, without using any stabilizing surface agent, make them suitable materials for catalytic applications. We employed the catalytic reduction of 2-nitroaniline (2-NA) to probe the catalytic activity of the nanostructures. Nitrobenzenes’ reductions are largely investigated in order to assess the catalytic activity of noble metal nanomaterials due to the simplicity in extracting kinetic data from spectroscopic measurements (Le et al., 2014; Hajfathalian et al., 2015; Menumerov et al., 2016; Naseem et al., 2017; Saire-Saire et al., 2019). In our case, we used 2-NA, which is an environmental pollutant and is the by-product of several anthropogenic chemical processes, such as the production of dyes, explosives and pharmaceutical products (Naseem et al., 2017). The product of its catalytic reduction is the ortho-phenylenediamine (o-PDA), a precursor for the synthesis of antioxidants, polymers, surfactants, and for products of food and fine chemistry industries. Therefore, the conversion of 2-NA into o-PDA is an environmental solution and a chemical opportunity.

**FIGURE 4:**
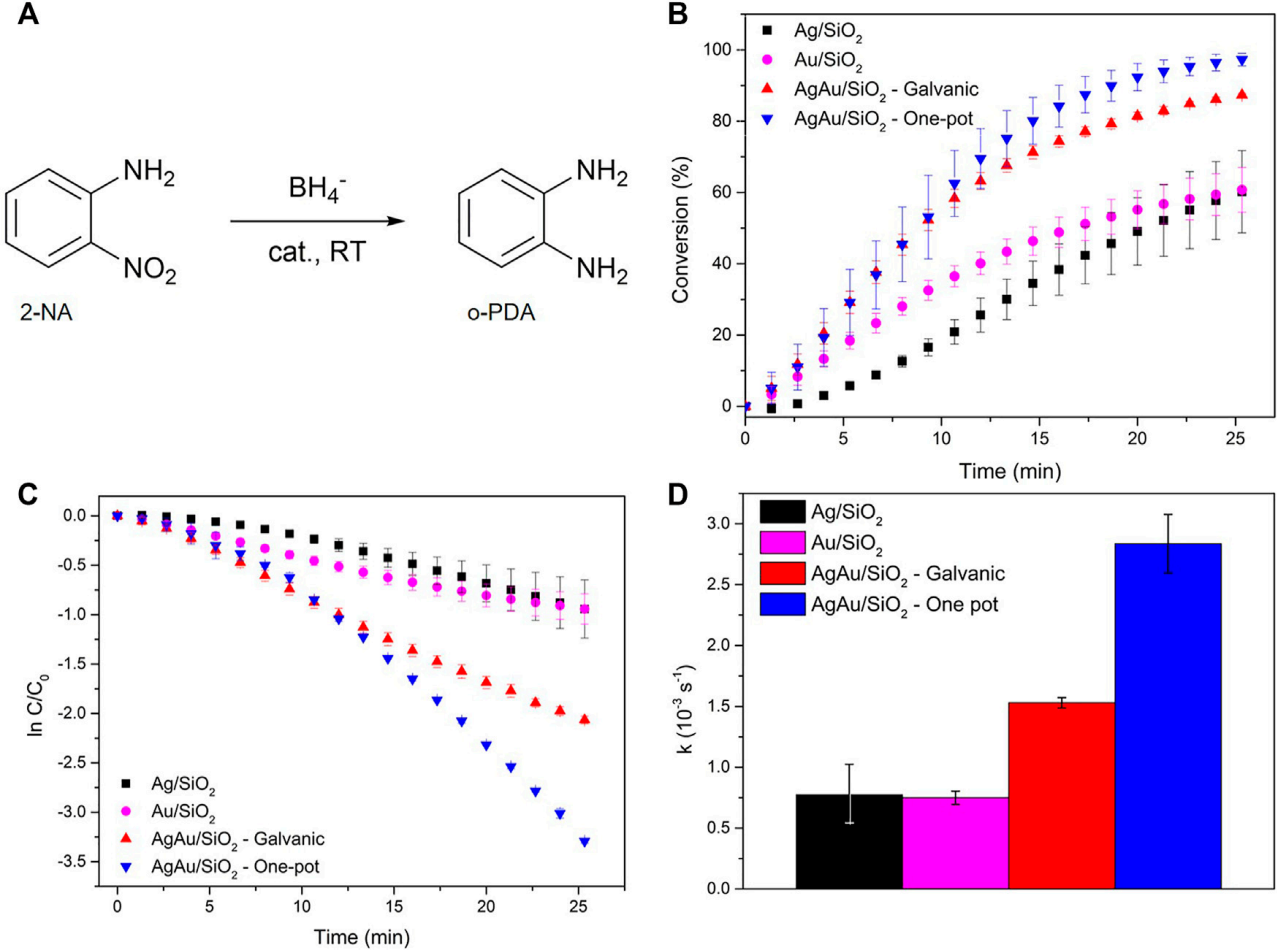
Catalytic reduction of 2-NA. Reaction scheme of 2-NA reduction to o-PDA **(A)**. Reduction of 2-NA analyzed by the decay of 412 nm band **(B)**. Linearization considering a pseudo first-order reaction **(C)**. Reaction pseudo first-order rate constant values obtained from C **(D)**.

The scheme of the catalytic reduction of 2-NA by BH_4_^−^ is illustrated in Figure 4A. The same amount of total metal load was used in each catalytic run of the different materials. The metal load was determined by ICP-OES (Supplementary Table S1). The conversion of 2-NA was followed by UV-VIS spectroscopy through the decay of 412 nm band, as demonstrated by Supplementary Figure S4. A shift in the 283—288 nm range was also observed throughout the catalytic reaction, and it is assigned to the formation of o-PDA (Supplementary Figure S5). As the catalytic reaction progresses, the yellowish starting solution characteristic of 2-NA becomes transparent with its conversion to o-PDA. Figure 4B show the triplicate of conversion curves for the different mechanochemically synthesized catalytic materials. The first points of these curves represent the induction period necessary for restructuring the surface of the catalyst with the substrate, just as observed for others nitrobenzenes (Hervés et al., 2012). This induction period plays a major role for Ag/SiO_2_, slowing up the 412 nm band decay for this material in the first minutes, whereas Au/SiO_2_ and both alloys show a shorter induction period. For the same period of reaction (∼25 min), the conversions were around 60% for both Ag/SiO_2_ and Au/SiO_2_. These values are much lower in comparison to the nanoalloys, which reached 2-NA conversions of 87 and 97%, for AgAu/SiO_2_ galvanic and AgAu/SiO_2_ one-pot, respectively.

The apparent reaction rate constants (*k*) were obtained after linearization of the conversion data as presented in Figure 4C, considering a typical pseudo-first order reaction (high excess of NaBH_4_) (Figure 4D). The induction periods were not considered for this calculation since they do not follow a linear trend. Similar apparent rate constants were shown by Ag/SiO_2_ (*k*_*Ag*_ = 7.75 10^–4^ *s* ^*−1*^) and Au/SiO_2_ (*k*_*Au*_ = 7.50 10^–4^ *s* ^*−1*^), values that are consistent with previously reported studies for monometallic Ag and Au nanomaterials in the catalytic reduction of 2-NA (Nalawade et al., 2013; Dong et al., 2014). However, AgAu/SiO_2_ galvanic and AgAu/SiO_2_ one-pot showed twice and four-fold the rate constant values of *k*_*Galvanic*_ = 1.53 10^–3^ *s* ^*−1*^ and *k*_*one-pot*_ = 2.84 10^–3^ *s* ^*−1*^, respectively.

It is worth discussing the better performance shown by the AgAu/SiO_2_ one-pot material. One can suggest that the composition, i.e., the Ag/Au ratio, can affect the catalytic activity. The metal content determined by ICP-OES (Supplementary Table S1) indicates that AgAu/SiO_2_ galvanic is composed by 58 *wt*% of Ag and 42 *wt*% of Au, whereas the one-pot sample consists of 36 *wt*% of Ag and 64 *wt*% of Au. However, the similar activities for single metal NPs indicate no significant effect of the metal in our experimental conditions. Another possibility for such difference of the catalytic activity between the nanoalloys could be the surface composition, as the studied catalytic reaction strongly depends on the surface chemistry. The surface composition estimated by XPS (Supplementary Table S3) shows the same fraction of Ag (62 *wt*%) and Au (38 *wt*%) for both galvanic and one-pot prepared nanostructures. Thus, this possibility solely can also be dismissed. A final aspect that must be considered is simply the difference of particle size. AgAu/SiO_2_ one-pot material presents smaller particles (4.0 nm) compared to the material prepared by the galvanic route (6.1 nm). It is of wide knowledge that smaller sizes are related to higher specific surface areas, which can be available for catalysis, and consequently increase the reaction rate.

Conversion, induction period and reaction rate constant strongly suggest that the bimetallic nanoalloys benefit from the synergy between silver and gold, just as previously reported (Wang et al., 2014; Kisukuri et al., 2016; da Silva et al., 2021). Although many examples of enhanced catalytic properties of bimetallic systems have been reported, the synergistic effect between metals in these systems is yet to be fully comprehended. Among reaction conditions such as pH, temperature and diffusion rates, catalytic rate constants are related to adsorption energy of reactants on active sites. In its turn, adsorption energy is dependent on the crystallite size, facets and composition, properties that are susceptible of changes by the formation of alloys in bimetallic systems. Besides, the rearranged atomic ordering admitted by alloys may change the surface electronic structure of the nanoparticles and, thus, affect their catalytic properties (Sha et al., 2019).

## 4 Conclusion

Bimetallic nanoparticles offer the possibility to combine the different properties in a single structure. We demonstrated different possibilities to prepare crystalline AgAu nanoalloys through bottom-up mechanochemical synthesis. Both galvanic and one-pot/co-reduction strategies led to the formation of nanoalloys with diameters of 6.1 ± 1.7 nm and 4.0 ± 1.0 nm, respectively. STEM-EDX images and XPS confirmed the formation of these alloys. The LSPR in the UV-VIS DRS, however, demonstrated that some monometallic NPs or regions of higher density of a single element can also be formed. This shows that further investigation into elementary steps of the nanostructure formation is necessary if one aims an effective control of the final materials, and therefore, their properties. Our results reinforces that we need to understand the specific rules for solid-state mechanochemical synthesis of nanomaterial from their respective precursors. This includes the different rates of reactions, and therefore, the nucleation and the formation of feeding species to the growing sites.

Additionally, in our work, the mechanochemical route was able to generate materials with efficient distribution over the support. Besides, the direct synthesis over SiO_2_ dismisses the use of capping agents and eliminate impregnation steps. The catalytic activity of our AgAu/SiO_2_ nanoalloys were tested in the reduction of 2-NA. The alloyed nanoparticles displayed higher catalytic activity in terms of reaction rate constants (*k*_*Galvanic*_ = 1.53 10^–3^ *s* ^*−1*^ and *k*_*one-pot*_ = 2.84 10^–3^ *s* ^*−1*^) and conversion (87 and 97%, respectively) when compared to the single metal NPs (*k*_*Ag*_ = 7.75 10^–4^ *s* ^*−1*^ and *k*_*Au*_ = 7.50 10^–4^ *s* ^*−1*^ with 60% conversion for both materials). The higher reaction rate and conversion were obtained for the AgAu/SiO_2_ one-pot material, showing the dominant effect of the particle size. It still worth mentioning that the LSPR displayed by the nanoalloys are very interesting for further studies on light-enhanced plasmonic catalysis.

The mechanochemical route for the preparation of multicomponent catalysts with active surfaces is highly envisioned to improve catalytic activity and minimize costly processes. This also includes the possibility of scaling up the process to meet industrial requirements, in addition of being a powerful tool for a sustainable design of new materials.

## Data Availability

The raw data supporting the conclusion of this article will be made available by the authors, without undue reservation.
