# Supplementary material for: Mechanochemical Strategies for the Preparation of SiO2-Supported AgAu Nanoalloy Catalysts
Source: Front Chem. 2022 Feb 2;10:836597. doi: 10.3389/fchem.2022.836597 (PMC8847606; doi:10.3389/fchem.2022.836597)
Supplement: Supplementary file 1 [file DataSheet1.pdf]

Supplementary Material

Mechanochemical Strategies for the Preparation of SiO_2 – Supported AgAu Nanoalloy Catalysts

Rafael T. P. da Silva[1], Susana I. Córdoba de Torresi[1], Paulo F. M. de Oliveira[1*]

[1]Institute of Chemistry, University of São Paulo - Brazil

*** Correspondence:**

Paulo F. M. de Oliveira
paulofmo@usp.br

Catalytic Reduction of 2-Nitroaniline S2

XPS Data Acquisition and Analysis S2

Supplementary Figure S1 – Particle size distribution of the the metallic nanoparticles S3

Supplementary Figure S2 – HAADF-STEM and STEM-EDX characterization of the bimetallic materials. S4

Supplementary Table S1 - ICP-OES analysis S4

Supplementary Table S2 - Reaction yield for one-hour milling……..S5

Supplementary Figure S3 - XPS spectra of the mechanochemically prepared samples. S6

Supplementary Table S3 – XPS elemental analysis S7

Supplementary Figure S4 – Catalytic reduction of 2-NA to o-PDA. S8

Supplementary Figure S5 – Band shift in the range of 280 nm throughout the catalytic reduction of 2-NA S9

Catalytic Reduction of 2-Nitroaniline

A 1 mg·mL^{-1} aqueous stock solution of each catalyst was prepared. In a cuvette, 2.8 mL of water, 120 µL of 2-NA (6.25 mM), 130 µL of $NaBH_4$ (2 mM) and, at last, the volume correspondent to 1.6 % (m/m) of metal/2-NA of each catalyst was added. The spectra were collected every 80 seconds. The determination of the reaction rate constant followed the pseudo first-order,

$$A = A_0 e^{-\frac{k}{t}} \quad \text{(S1)}$$

in which A = A_{412}-A_{500}, A_0 = initial absorbance at 412 nm, k is the reaction rate constant and t is time. Each reaction was repeated three times. Between catalytic runs, the cuvette was washed with aqua regia and several times with water to eliminate any deposition of metals.

The conversion (%) was calculated by:

$$Conversion\ (\%) = \frac{A_0 - A}{A_0} \times 100 \quad \text{(S2)}$$

Considering Equation S1 and a pseudo-first order reaction, the linearization of the conversion curves followed:

$$\ln\left(\frac{C}{C_0}\right) = ln\left(\frac{A}{A_0}\right) = -kt \quad \text{(S3)}$$

XPS Data Acquisition and Analysis

XPS spectra were collected using Mg Kα line as a source of ionization (hν = 1256.6 eV) and the pass energy was adjusted to 15 eV. The inelastic scattering of XPS of Ag 3d and Au 4f were subtracted using Shirley's method. Composition of the surface (<5 nm) was determined by the relative proportional areas of the peaks corrected by the Scofield atomic sensitivity factor with a precision of ±5%. Binding energy were corrected using the hydrocarbon components (C-H) set at 284.9 eV. Spectra were deconvoluted using a Voigtian function with a convolution of Gaussian (70%) and Lorentzian (30%) functions. FWHM varied between 1.2 and 2.1 eV and the peak positions were determined with a precision of ± 0.1 eV.

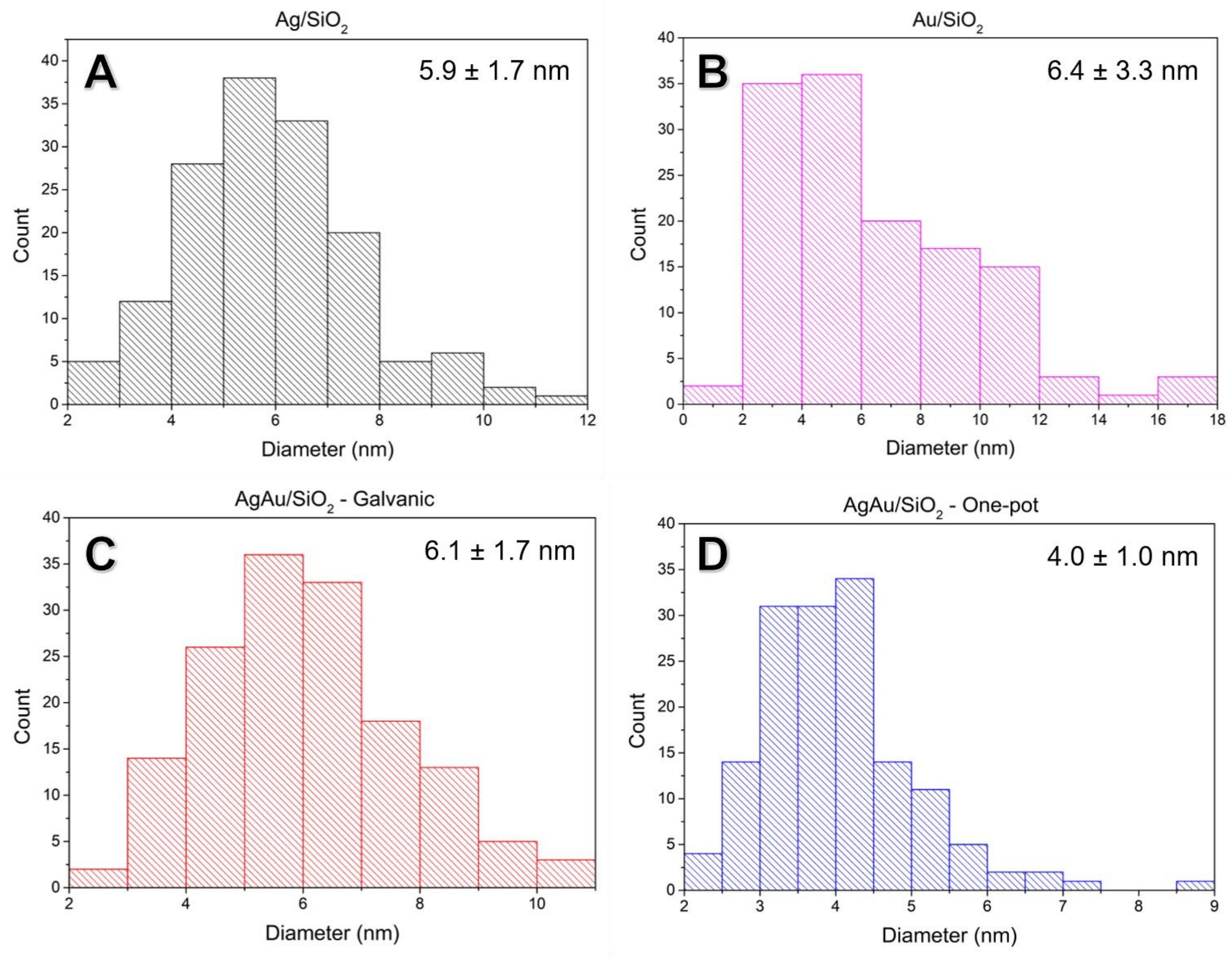

Supplementary Figure S1 – Particle size distribution of the the metallic nanoparticles on the synthesized materials. Ag/SiO_2 **(A)**, Au/SiO_2 **(B)**, $AgAu/SiO_2$ galvanic **(C)**, $AgAu/SiO_2$ one-pot **(D)**. For particle size distribution at least 150 individual particles were considered.

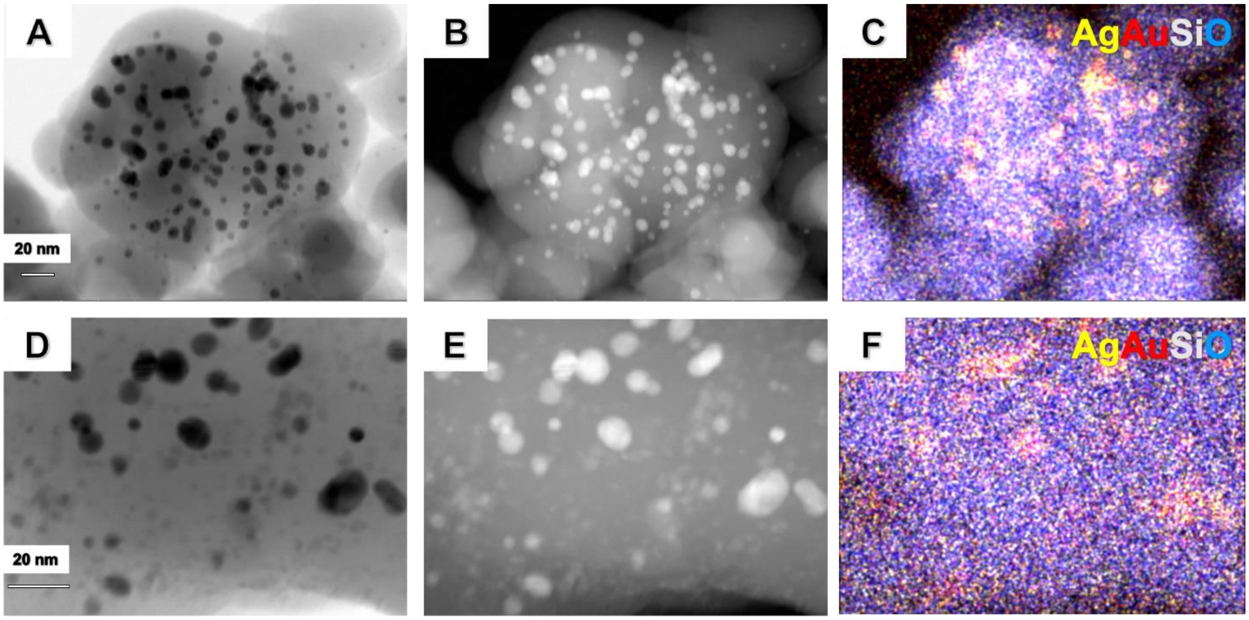

Supplementary Figure S2 – HAADF-STEM and STEM-EDX characterization of the bimetallic materials. AgAu/SiO_2 galvanic **(A-C)**, AgAu/SiO_2 one-pot **(D-F)**.

Supplementary Table S1 - ICP-OES analysis

Sample	Total metal in sample (mass %)	Ag (mass %)	Au (mass %)
Ag/SiO_2	2.50	100	-
Au/SiO_2	2.84	-	100
AgAu/SiO_2 galvanic	3.10	58	42
AgAu/SiO_2 one-pot	2.73	36	64

Supplementary Table S2 – Reaction yield for 1h-milling calculated from ICP-OES and metal salt added.

Sample	Initial amount of Ag (mass %)	Initial amount of Au (mass %)	Obtained amount of Ag (mass %)	Obtained amount of Au (mass %)	Yield Ag (%)	Yield Au (%)
Ag/SiO_2	4.41	-	2.50	-	56.7	-
Au/SiO_2	-	4.60	-	2.84	-	61.7
$AgAu/SiO_2$ galvanic	2.50	1.60	1.80	1.29	72.0	80.6
$AgAu/SiO_2$ one-pot	2.11	2.34	0.99	1.75	46.9	74.8

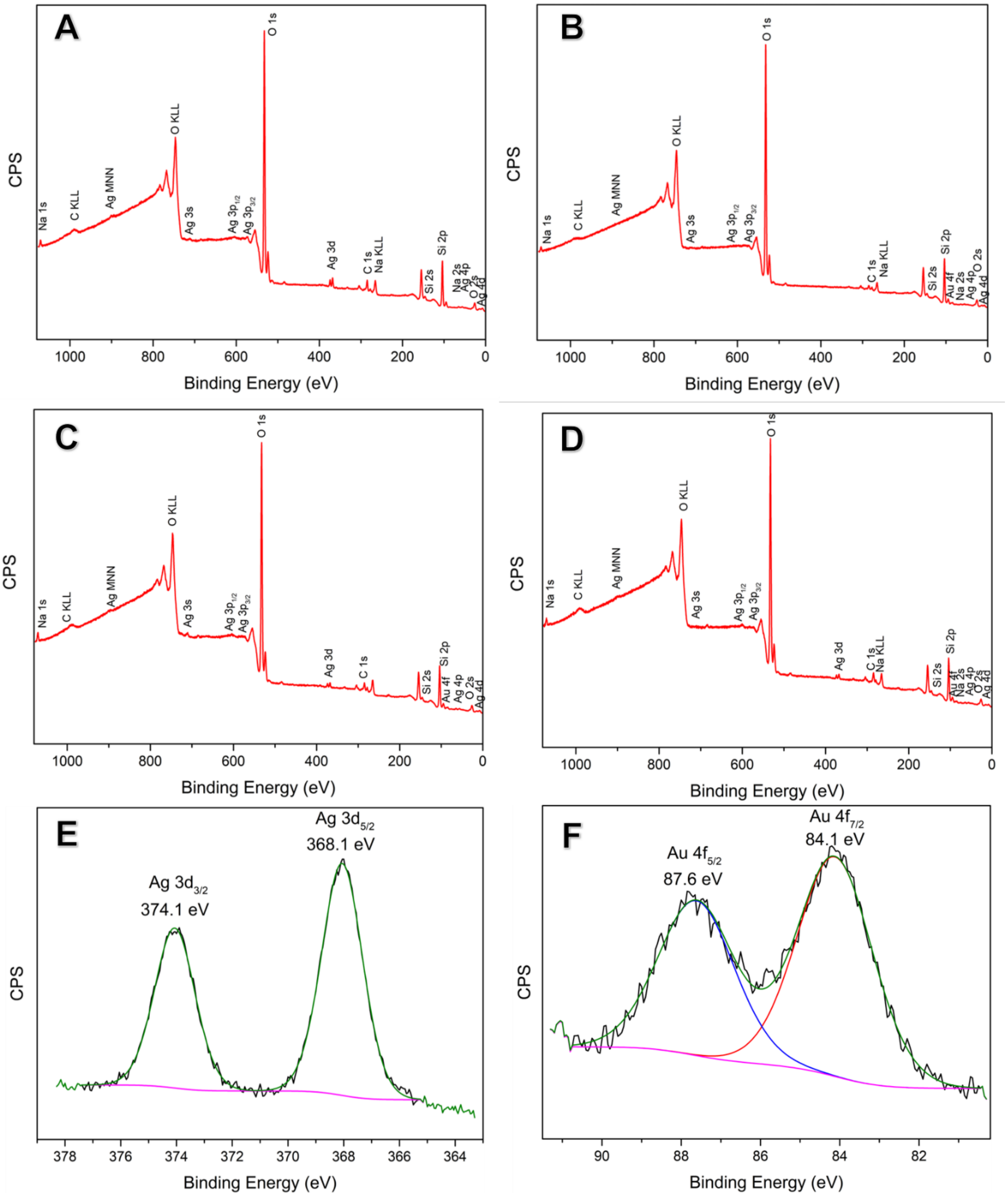

Supplementary Figure S3 - XPS spectra of the mechanochemically prepared samples. Survey spectra of Ag/SiO_2 **(A)**, Au/SiO_2 **(B)**, $AgAu/SiO_2$ Galvanic **(C)**, $AgAu/SiO_2$ one-pot **(D)**. HR-XPS for Ag 3d of Ag/SiO_2 **(E)** and Au 4f of Au/SiO_2 **(F)** regions.

Supplementary Table S3 – XPS elemental analysis

Sample	Ag (mass %)	Au (mass %)
Ag/SiO_2	100	-
Au/SiO_2	-	100
AgAu/SiO_2 galvanic	62	38
AgAu/SiO_2 one-pot	62	38

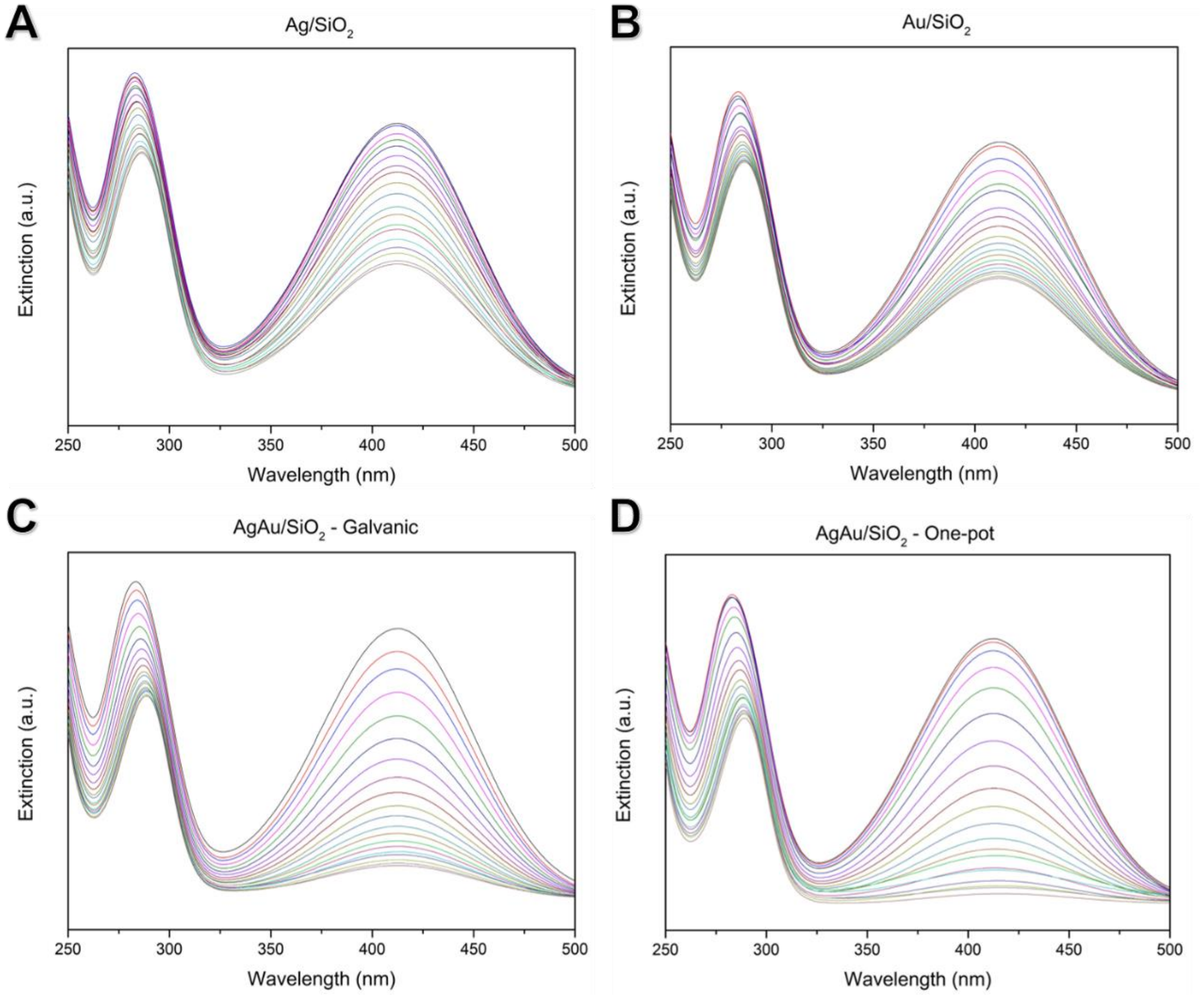

Supplementary Figure S4 – Catalytic reduction of 2-NA to o-PDA with $NaBH_4$ catalyzed by Ag/SiO_2 **(A)**, Au/SiO_2 **(B)**, $AgAu/SiO_2$ galvanic **(C)** and $AgAu/SiO_2$ one-pot **(D)**.

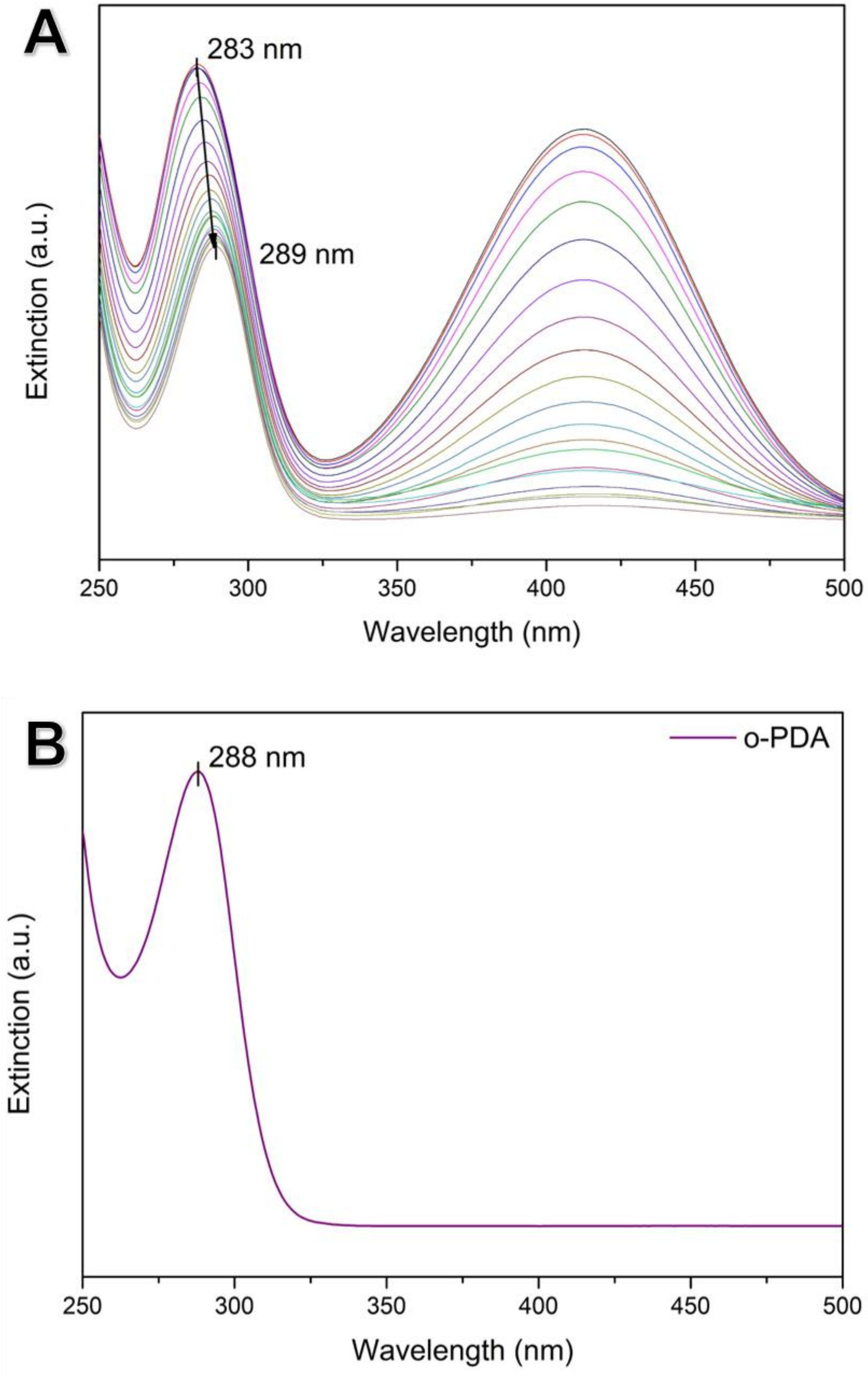

Supplementary Figure S5 – Band shift in the range of 280 nm throughout the catalytic reduction of 2-NA **(A)** represents the formation of o-PDA **(B).**
